# A data-driven epidemiological prediction method for dengue outbreaks using local and remote sensing data

**DOI:** 10.1186/1472-6947-12-124

**Published:** 2012-11-05

**Authors:** Anna L Buczak, Phillip T Koshute, Steven M Babin, Brian H Feighner, Sheryl H Lewis

**Affiliations:** 1 Johns Hopkins University Applied Physics Laboratory, 11100 Johns Hopkins Rd, Laurel, MD 20723-6099, USA

**Keywords:** Dengue fever, Prediction, Association rule mining, Fuzzy logic, Predictor variables

## Abstract

**Background:**

Dengue is the most common arboviral disease of humans, with more than one third of the world’s population at risk. Accurate prediction of dengue outbreaks may lead to public health interventions that mitigate the effect of the disease. Predicting infectious disease outbreaks is a challenging task; truly predictive methods are still in their infancy.

**Methods:**

We describe a novel prediction method utilizing Fuzzy Association Rule Mining to extract relationships between clinical, meteorological, climatic, and socio-political data from Peru. These relationships are in the form of rules. The best set of rules is automatically chosen and forms a classifier. That classifier is then used to predict future dengue incidence as either ***HIGH*** (outbreak) or ***LOW*** (no outbreak), where these values are defined as being above and below the mean previous dengue incidence plus two standard deviations, respectively.

**Results:**

Our automated method built three different fuzzy association rule models. Using the first two weekly models, we predicted dengue incidence three and four weeks in advance, respectively. The third prediction encompassed a four-week period, specifically four to seven weeks from time of prediction. Using previously unused test data for the period 4–7 weeks from time of prediction yielded a positive predictive value of 0.686, a negative predictive value of 0.976, a sensitivity of 0.615, and a specificity of 0.982.

**Conclusions:**

We have developed a novel approach for dengue outbreak prediction. The method is general, could be extended for use in any geographical region, and has the potential to be extended to other environmentally influenced infections. The variables used in our method are widely available for most, if not all countries, enhancing the generalizability of our method.

## Background

Dengue is an acute febrile disease of humans caused by a single-stranded RNA flavivirus transmitted by *Aedes* mosquitoes, primarily *Aedes aegypti*. These mosquitoes thrive in tropical urban areas by breeding in uncovered containers capable of holding rain water, such as tires, buckets, flower pots, etc. [1]. Dengue is now the most common arboviral disease of humans in the world [2,3], recognized in over 100 countries, with an estimated 50 – 100 million cases annually [4,5]. More than one third of the world’s population lives in the areas where there is a risk of dengue virus transmission. Recent dengue outbreaks have occurred in the Philippines, Singapore, Thailand, Cambodia, Peru, Ecuador, and Brazil [6]. Dengue is endemic in Puerto Rico and recently re-emerged in the Florida Keys in the United States (US) [7].

Dengue presents with a wide range of symptoms [2]. Minimally symptomatic or mild flu-like presentations may be seen in young children. The classic presentation (called dengue fever or DF), seen most commonly in older children and adults, is an abrupt onset of a high fever, severe muscle and joint pain, and headache that may occur with nausea and vomiting. Recovery is prolonged and marked by fatigue and depression [8]. A hemorrhagic form of the disease may develop, especially in patients who have been exposed to more than one of the four known strains of the virus [2,6]. This presentation, called dengue hemorrhagic fever (DHF), includes increased capillary permeability with potentially significant vascular leakage that compromises organ function and may lead to shock [2-4]. Mortality in DHF with excellent medical care is generally less than 10%, but has been reported to be as high as 40% in austere settings [2].

Efforts to develop a dengue vaccine have been hampered by lack of appropriate animal models. Additionally, the empirical observation of increased incidence of DHF with prior immunologic response to dengue virus infection raises the theoretical possibility that immunization may result in an increased incidence of DHF [9]. Several dengue vaccines are currently undergoing clinical trials; however, no dengue vaccine is licensed for use in the US. Therefore, it is important to find ways to accurately predict dengue outbreaks in order that preventive public health interventions may be used to mitigate the effect of these outbreaks, particularly in areas where resources for such efforts are limited and where medical treatment facilities may become overwhelmed by an outbreak.

## Methods

### Predictor variables

Previous investigators have described dengue predictive models using a variety of different input variables [10-19]. Because of characteristics of vector transmission, pre-outbreak dengue incidence rate [1] and seroprevalence [20] reflect the presence of the virus in the human population and are expected to be good indicators of outbreak potential. Temperature is also often used because of its effects on biological parameters such as the extrinsic incubation period of the mosquito [1,21]. As the mosquito vector requires water for completion of its life cycle, investigators have examined the use of rainfall data for disease outbreak prediction [1]. These rainfall data may be locally acquired or derived via satellite measurements. The Tropical Rainfall Measuring Mission (TRMM) satellite data have been used to derive rainfall measurements [22] in remote and resource-limited regions and these measurements have been used for predictions for disease outbreaks [23]. In addition, satellite measurements of leaf area indices have been used to assess green leaf biomass, photosynthetic activity, and the effects of seasonal rainfall, which are then related to vector habitat characteristics and disease outbreaks [24]. Commonly used leaf area indices are the Normalized Difference Vegetation Index (NDVI) and the Enhanced Vegetation Index (EVI), both available from satellite sensors such as the Advanced Very High Resolution Radiometer (AVHRR) and the Moderate Resolution Imaging Spectrometer (MODIS). NDVI is closely related to photosynthesis, while EVI is closely related to leaf display [22]. Because climate effects, such as the El Nino Southern Oscillation (ENSO), can indicate near-term future rainfall anomalies, the Southern Oscillation Index (SOI) and various sea surface temperature anomalies (SSTA) have also been used as indicators of future disease outbreaks [10,25-27]. Our method uses variables such as previous dengue incidence, meteorological/climatic data (rainfall, day temperature, night temperature, NDVI, EVI, SSTA, SOI), and socio-economic data (political stability, sanitation, water, and electricity). Sources of these data can be found in Table 1.

**Table 1:** Sources of data

**Data type**	**Source**
Rainfall	NASA Tropical Rainfall Measuring Mission http://mirador.gsfc.nasa.gov/
Temperature	USGS Land Processes Distributed Active Archive Center https://lpdaac.usgs.gov/get_data
Altitude	NOAA National Geophysical Data Center http://www.ngdc.noaa.gov/cgi-bin/mgg/ff/nph-newform.pl/mgg/topo/.
Demographics	Peru National Institute of Statistics and Information http://www.inei.gob.pe/
NDVI	USGS Land Processes Distributed Active Archive Center https://lpdaac.usgs.gov/get_data
EVI	USGS Land Processes Distributed Active Archive Center https://lpdaac.usgs.gov/get_data
Political Stability	Worldwide Governance Indicators Project http://info.worldbank.org/governance/wgi/index.asp
Southern Oscillation Index	US National Center for Atmospheric Research http://mirador.gsfc.nasa.gov/
Sea Surf. Temp. Anomaly	NASA Global Change Mastery Directory https://lpdaac.usgs.gov/get_data

In order to perform spatiotemporal predictions, all the variables need to fit the same spatiotemporal scale. The spatiotemporal scale used in this work was selected based on the distribution of the dengue data: the chosen temporal scale was one week and the chosen spatial distribution was one district. In the following sections, when describing the different variables used, we also describe the extensive preprocessing done for each variable to fit the selected spatiotemporal scale.

#### Dengue case data

**Figure 1:**
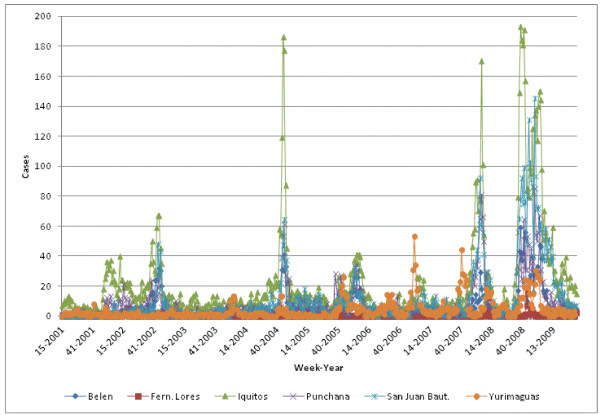
Dengue cases per week.

We obtained dengue case data from our collaborators at the Peruvian Ministry of Health. We took into account cases marked as “probable” and “confirmed” and did not include cases labeled “discarded.” Information for each case included year, week number (within a year), and district. With this information, cases per week in a given district could be counted (Figure 1). The data set included dengue case data from 2001–2009.

**Figure 2:**
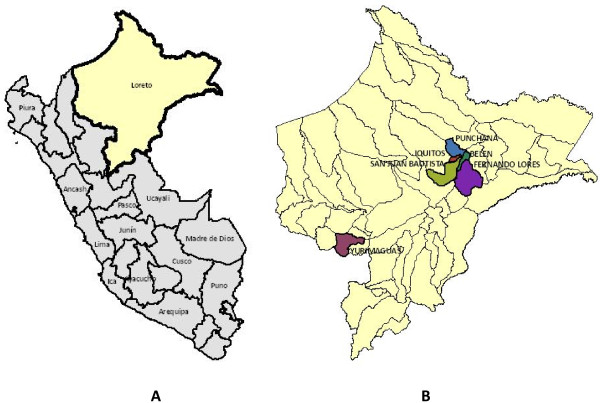
**(A) Departments in Peru.** We used Loreto (shaded in yellow) for our study. (**B**) Fifty-one districts in the Loreto department. We used six districts for analysis (shaded in darker colors).

The province of Loreto, from which we had these data, consists of 51 districts. In this study, we considered only six of those districts (see Figure 2) that had a large number of dengue cases (Belen, Fernando Lores, Iquitos, Punchana, San Juan Bautista, and Yurimaguas).

**Figure 3:**
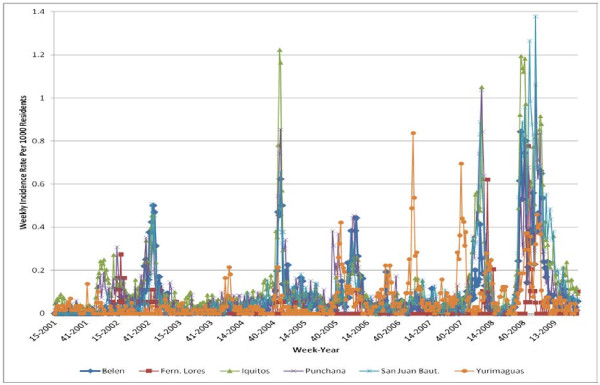
**Weekly dengue *Incidence Rate *per 1000 residents.**

For a given district, we calculated dengue incidence per week per 1000 residents (Figure 3):

$${\text{Incidence}}_{\text{week}}=♯{\text{cases}}_{\text{week}}*1000/{\text{population}}_{\text{week}}$$

In the calculations, we assumed that the population of a district was constant throughout a given year. To derive these population values, we obtained district population data from Peru National Institute of Statistics and Information from the 1993 and 2007 censuses (there was no census taken in between these years). For each district, we used a linear interpolation to obtain the population for each of the years between 1993 and 2007, and we used a linear extrapolation to obtain the population for 2008 and 2009. When portions of Iquitos were reassigned to Belen and San Juan Bautista in 2000, we assumed that the three districts’ total population increased linearly and that the ratio between them remained constant.

**Figure 4:**
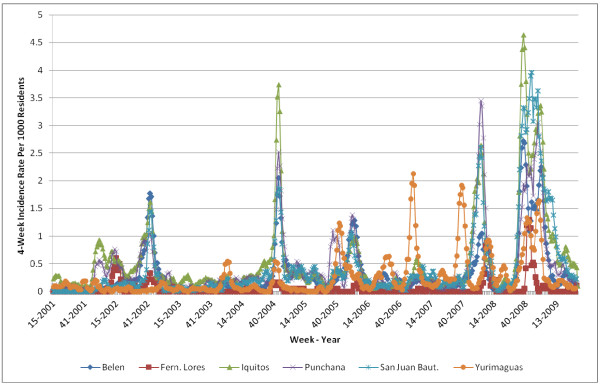
**4-Week dengue *Incidence Rate *per 1000 residents.**

For subsequent analysis, we aggregated weekly incidence values into four-week interval values (Figure 4), by adding the individual weeks’ incidence values. Specifically, the incidence for the interval from week *i* to week *i* + 3 can be obtained with:

$${\text{Incidence}}_{\text{i}-\text{i}+3}={\text{Incidence}}_{\text{i}}+{\text{Incidence}}_{\text{i}+1}+{\text{Incidence}}_{\text{i}+2}+{\text{Incidence}}_{\text{i}+3}$$

Because dengue cases were provided in weekly intervals, we converted all other input variables to weekly intervals. Following US Centers for Disease Control and Prevention (CDC) conventions [28], all weekly intervals begin on a Sunday.

#### Rainfall

**Figure 5:**
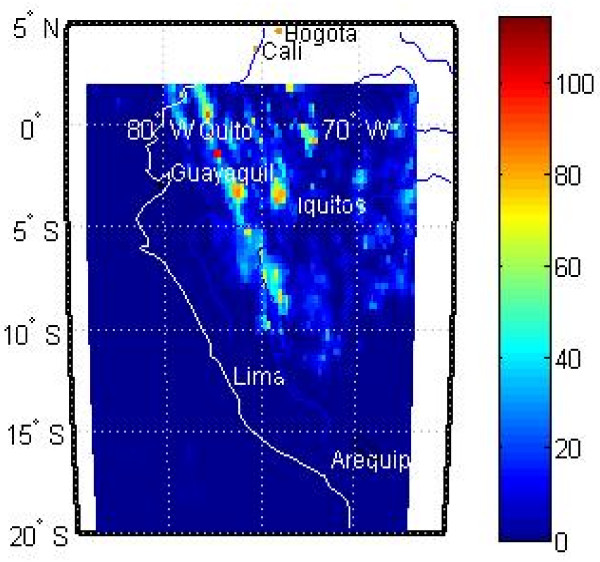
**Example of satellite-derived daily rainfall for Peru.** Units are mm.

Rainfall data with 0.25-degree resolution were obtained from the satellite measurements of the NASA Tropical Rainfall Measuring Mission (TRMM). After downloading data from the TRMM website [29] in hierarchical data format (HDF), we used MATLAB [30], tools to extract the relevant data layers. These data contained hourly rainfall rates, averaged over three-hour intervals. To convert from rainfall rates to rainfall amounts, we multiplied all data by three (the number of hours in the measurement interval). We then aggregated the resulting data into daily and weekly totals. Following CDC convention [28], we defined all weeks to begin on a Sunday. Figure 5 shows an example of rainfall amounts for a single day. In the data set, some data were missing so we assigned rainfall totals of zero for each of these instances.

**Figure 6:**
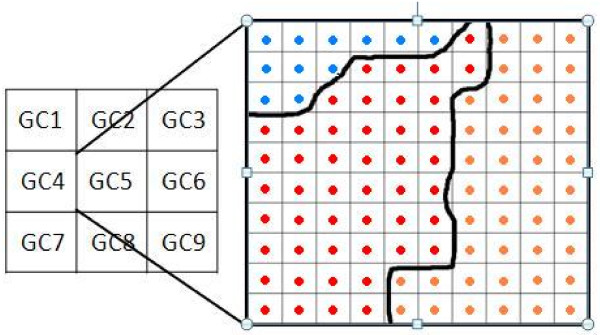
Example of dividing a grid cell into subcells and counting the centroids falling within each district.

Spatial aggregation of rainfall data from 0.25 degree grid cells to districts was performed by first computing a weight equivalent to estimated proportions of a district comprised by each grid cell. We then used each district’s set of weights and the gridded rainfall values for a given week, to determine a single rainfall amount for each district during each week. To compute the weight for a given district, we evenly divided each 0.25 degree cell into 100 *subcells* and counted (using MATLAB [30]) the number of centroids of these subcells contained within that district. We then divided these counts by the total number of subcell centroids encompassed by each district. An example is shown in Figure 6: 11 subcell centroids fell within district A (shown in blue), 47 within district B (red), and 42 within district C (orange). Table 2 gives counts of subcell centroids in each district in other grid cells (not shown). For some of the cells, the total of subcell centroids is less than 100 because other districts (not listed) encompass some of the subcell centroids in those grid cells. The following calculations can be used to determine the weights *W*_*i,d*_ for each grid cell for district A:

$${\text{W}}_{1,\text{A}}=7/52=0.13$$

$${\text{W}}_{2,\text{A}}=22/52=0.42$$

$${\text{W}}_{4,\text{A}}=12/52=0.23$$

$${\text{W}}_{5,\text{A}}=11/52=0.22$$

$${\text{W}}_{3,\text{A}}={\text{W}}_{6,\text{A}}={\text{W}}_{7,\text{A}}={\text{W}}_{8,\text{A}}={\text{W}}_{9,\text{A}}=0$$

**Table 2:** Numbers of subcell centroids from each grid cell in districts A, B, and C. GC stands for Grid Cell

**District**	**GC1**	**GC2**	**GC3**	**GC4**	**GC5**	**GC6**	**GC7**	**GC8**	**GC9**
A	7	22	0	12	11	0	0	0	0
B	0	2	0	67	47	0	18	13	0
C	0	5	15	0	42	34	0	65	25

Similar calculations were performed to determine the grid cell weights for other districts and the same technique was used to convert other variables’ gridded data to single value for each district.

For subsequent analysis, we aggregated weekly rainfall values into four-week interval values, by adding the individual weeks’ rainfall values. Specifically, the rainfall for the interval from week *i* to week *i* + 3 can be obtained with:

$$Rainfall_{i-i+3}=Rainfall_{i}+Rainfall_{i+1}+Rainfall_{i+2}+Rainfall_{i+3}$$

#### Temperature

**Figure 7:**
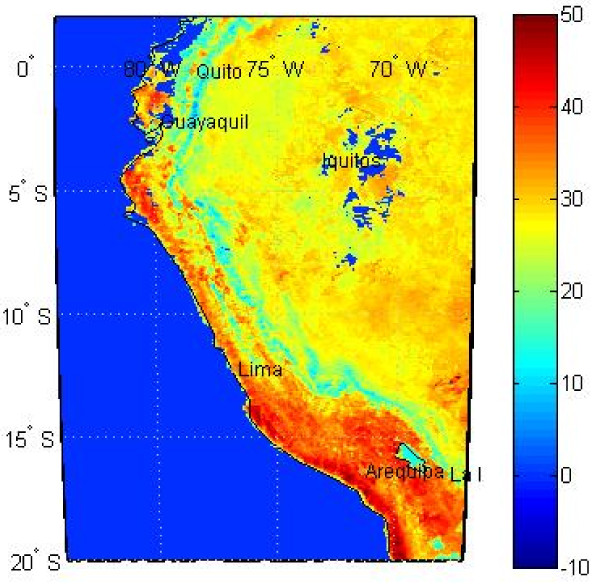
**Example *of day-time temperature *data for a given 8-day interval.** Units are degrees Celsius. Procedures were developed in MATLAB to remove missing data. The dark blue spots near Iquitos (corresponding to a temperature near 0 C) were removed since they were corresponding to missing data.

We obtained eight-day interval temperature means with 0.05 degree resolution from the United States Geological Survey (USGS) Land Processes Distributed Active Archive Center. After downloading the data from their website [31] in HDF file format, we used MATLAB [30] tools to extract the relevant data layers. We considered and identically processed both daytime and nighttime temperatures. An example of daytime temperature values for a given 8-day interval are shown in Figure 7.

**Figure 8:**
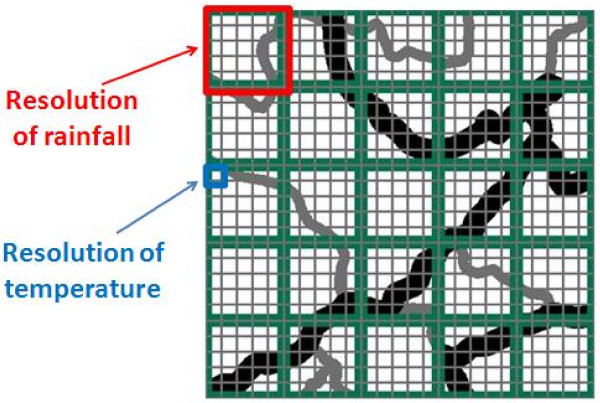
Illustration of spatial resolution of different variables.

We converted the temperature data from 0.05 degree to 0.25 degree resolution in order to match the resolution of the rainfall data (shown in Figure 8). Generally, we aggregated the data into the quarter-degree cells by averaging the 25 smaller cells’ values. But where some of the data were missing, we only included the grid cells with actual data. If all temperature values were missing for an entire 0.25 degree grid cell, we set the temperature value for that grid cell to “missing”.

Subsequently, we calculated single-week averages from eight-day means, coincident with weekly dengue incidence data. In some cases, an entire week was contained with an 8-day interval and we set the temperature for that week to the means from that 8-day interval. In other cases, we used weighted sums of the means of adjacent 8-day intervals. Specifically, we applied:

$$T_{\mathrm{week}}=T_{8,i}\left(d_{i}/7\right)+T_{8,i+1}\left(d_{i+1}/7\right)$$

where *d*_*i*_ is the number of days in the week overlapping the *i*th 8-day interval and *T*_*8,i*_ is the temperature in that 8-day interval.

In cases where one of two 8-day intervals had missing data from a given grid cell, we used the mean from the other 8-day interval exclusively. In cells where all values from corresponding 8-day intervals were “missing” data, we excluded these temperature values from the subsequent spatial aggregation whenever possible. (In a small number of cases, all temperature data from all grid cells comprising a given district were missing for an entire week and we set that district’s temperature to “missing”). To determine grid cell weights for each district, we applied the subcell centroid counting method (described earlier in the section entitled ***Rainfall***) that we used to determine grid cell weights for rainfall.

For subsequent analysis, we aggregated weekly temperature values into four-week interval values, by taking the average of the individual weeks’ temperature values. Specifically, the temperature for the interval from week *i* to week *i* + 3 can be obtained with:

$$Temperature_{i\_i+3}=\left(Temperature_{i}+Temperature_{i+1}+Temperature_{i+2}+Tempearture_{i+3}\right)/4$$

#### Vegetation indices: NDVI and EVI

**Figure 9:**
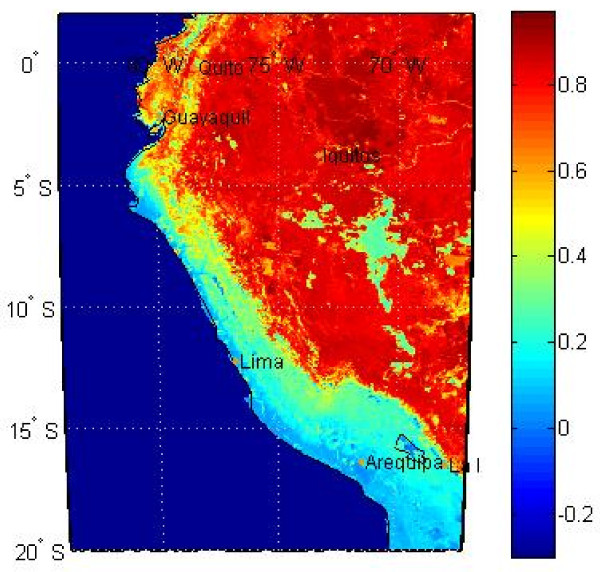
**Example of *NDVI *values for a given 16-day interval.**

**Figure 10:**
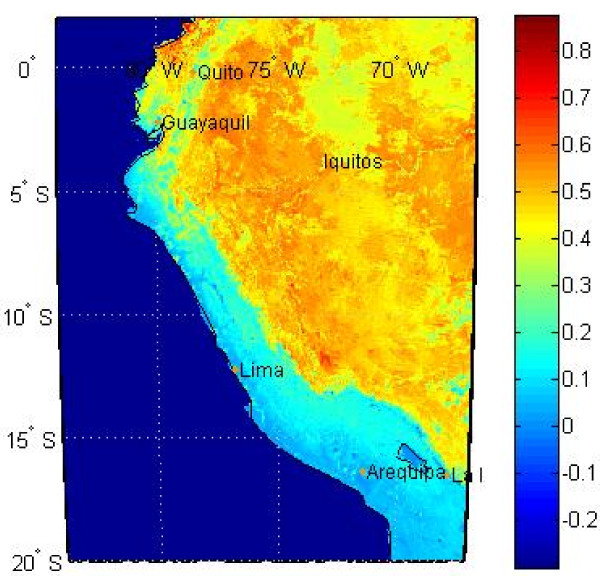
**Example of *EVI *values for a given 16-day interval.**

We obtained 16-day interval Normalized Difference Vegetation Index (NDVI) values and Enhanced Vegetation Index (EVI) values with 0.05 degree resolution from the USGS Land Processes Distributed Active Archive Center [31]. Examples of NDVI and EVI data are shown in Figures 9 and Figure 10, respectively.

These data consist of satellite measurements of leaf area indices that provide a surrogate assessment of green leaf biomass, photosynthetic activity, and the effects of seasonal rainfall, which may then be related to vector habitat characteristics and disease outbreaks [32]. NDVI is closely related to photosynthesis, while EVI is closely related to leaf display [33]. Values of both NDVI and EVI were obtained from the Moderate Resolution Imaging Spectrometer (MODIS).

Negative values (approaching −1) of NDVI correspond to water. Values close to zero (−0.1 to 0.1) correspond to barren areas of rock, sand, or snow. Low positive values (approximately 0.2 to 0.4) represent shrub and grassland. High values (approaching 1) indicate temperate and tropical rainforests. NDVI seasonal variations closely follow human-induced patterns, resulting in a significant correlation between NDVI and landscape disturbance [33].

EVI is an optimized index designed to enhance the vegetation signal with improved sensitivity in high biomass regions and improved vegetation monitoring through a decoupling of the canopy background signal and a reduction in atmosphere influences. EVI is calculated similarly to NDVI, but corrects for some distortions in the reflected light. EVI is considered to be more responsive than NDVI to canopy structural variations. Xiao et al. [34] note that the fact that EVI includes the blue band for atmospheric correction is particularly important for the Amazon basin where seasonal burning of pasture and forest takes place throughout the dry season. They note that, unlike EVI, NDVI could be substantially impacted by the smoke and aerosols from biomass burning, regardless of the vegetation changes.

For NDVI and EVI, we used data processing steps similar to that of temperature: downloading in HDF format from the USGS LPDAAC website [31], extracting relevant data layers with MATLAB [30] tools, converting from 0.05 degree to 0.25 degree resolution, and accounting for missing data. Subsequently, we combined 16-day means to obtain single-week averages, coincident with weekly dengue incidence. In some cases, an entire week was contained with a 16-day interval and we set the NDVI and EVI values for that week to the values from that 16-day interval. In other cases, we used weighted sums of the values of adjacent 16-day intervals. Specifically, we applied:

$$N_{\mathrm{week}}=N_{16,i}\left(d_{i}/7\right)+N_{16,i+1}\left(d_{i+1}/7\right)$$

where *d*_*i*_ is the number of days in the week overlapping the *i*th 16-day interval and *N*_*16,i*_ is the NDVI or EVI value for that 16-day interval.

In cases where one of two 16-day intervals was missing data from a given grid cell, we used the value from the other 16-day interval exclusively. In cells where all values from corresponding 16-day intervals were missing data, we excluded these NDVI and EVI values from the subsequent spatial aggregation. To assign single NDVI/EVI values for each district, we applied the subcell centroid counting method described earlier in the section entitled ***Rainfall***.

For subsequent analysis, we aggregated weekly NDVI/EVI values into four-week interval values, by taking the average of the individual weeks’ NDVI/EVI values. Specifically, the NDVI/EVI for the interval from week *i* to week *i* + 3 can be obtained with:

$$Index_{i\_i+3}=\left(Index_{i}+Index_{i+1}+Index_{i+2}+Index_{i+3}\right)/4$$

where Index stands for NDVI or EVI depending for which one the calculation is being performed.

#### Southern Oscillation Index

We obtained monthly Southern Oscillation Index (SOI) values from the US National Center for Atmospheric Research Climate Analysis Section website [35]. SOI is based on the pressure difference between Darwin (Australia) and Tahiti (French Polynesia), which influences the strength of the prevailing easterly winds. These data provide a measure of the El Nino Southern Oscillation (ENSO) climate effect. A single monthly SOI value is available and therefore is not location-specific.

We processed monthly SOI values to obtain single-week values, coincident with weekly dengue data. In most cases, an entire week was contained with a given month and we set the SOI values for that week to the value from the encompassing month. In other cases, we used weighted sums of the values of adjacent month. Specifically, we applied:

$$S_{\mathrm{week}}=S_{m,i}\left(d_{i}/7\right)+S_{m,i+1}\left(d_{i+1}/7\right)$$

where *d*_*i*_ is the number of days in the week overlapping the *i*th month and *S*_*m,i*_ is the SOI for that month.

#### Sea Surface Temperature Anomaly

As a complement to SOI values, we obtained weekly Sea Surface Temperature Anomaly (SSTA) values from the NASA Global Change Mastery Directory website [36]. Different SSTA values are computed for different regions of the Pacific Ocean and we used the SSTA values for the regions directly adjacent to Peru (Nino regions 1 and 2). Unlike SOI, SSTA values are typically published for a single week, beginning on Wednesday. To align these values with weekly dengue data (beginning on Sunday), we computed weighted sums according to

$$A_{\mathrm{week}}=A_{w,i}\left(d_{i}/7\right)+A_{w},\left(d_{i+1}/7\right)$$

where *d*_*i*_ is the number of days in the week overlapping the *i*th week and *A*_*w,i*_ is the SSTA for that week.

#### Socio-economic and demographic data

We considered several socio-economic variables that reflected potentially relevant information. We obtained political stability data from the Worldwide Governance Indicators Project [37]. These data consisted of a single value for Peru from most years between 1996 and 2009. To obtain values for the missing years, we performed a linear interpolation. From the Peru National Institute of Statistics and Information 2007 census [38], we obtained population density and proportions with electric lighting, running water, and hygienic services. These data also included numbers of *vivendas particulares* (private dwellings), *vivendas con abstecimiento de agua* (private dwellings with running water), *vivendas con servicio higienico* (private dwellings with toilets), and *vivendas con alumbrado electric* (private dwellings with electricity) for each district. We then calculated percentages of private dwellings with running water, toilets, and electricity. Because these values were only available from the 2007 census, we used a single value for each district for all weeks.

#### Elevation

We obtained elevation data from the NOAA National Geophysical Data Center website [39]. We assigned missing data (typically for ocean locations) an elevation of zero. By averaging the elevation in 30-by-30 grids, we changed the scale from 1/120 degree to 0.25 degree resolution, consistent with the scale of rainfall data. Subsequently, we used the subcell centroid counting method (described earlier in the section entitled ***Rainfall***) to determine a single average elevation value for each district.

### Prediction methodology

#### Overview

**Figure 11:**
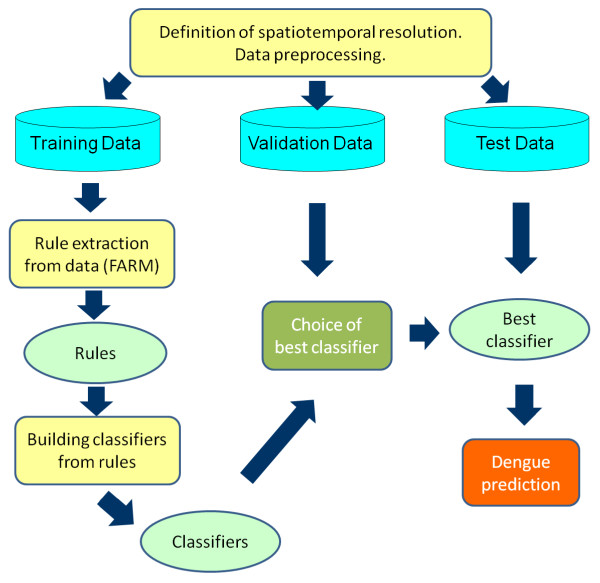
Dengue prediction method developed.

The dengue prediction methodology developed has the following steps (Figure 11):

1 Definition of spatiotemporal resolution and data preprocessing to fit that resolution.

2 Division of the data set into disjoint training, validation and test subsets.

3 Rule extraction from training data using Fuzzy Association Rule Mining (FARM).

4 Automatic building of classifiers from the rules extracted in step 3.

5 Choice of the best classifier based on its performance on the validation data set.

6 Computation of predictions on the test data using the classifier from step 5. Computation of performance metrics.

Because different model input data come in disparate spatiotemporal scales, they were converted to one spatiotemporal scale to be used in the prediction method. The chosen temporal scale was one week and the chosen spatial distribution was one district. In step 1 all the predictor and epidemiological data were converted into this spatiotemporal scale. Details of this conversion were described earlier in the section entitled **Predictor variables**.

The second step was to divide the data set into disjoint training, validation and test subsets. FARM [40] was used on the training subset in step 3 to extract rules predicting future dengue incidence (details described later in the section entitled **Association rule mining and fuzzy association rule mining**). Step 4 involved the automatic building of classifiers from rules extracted in step 3. A separate, validation subset was used to choose the best performing classifier in step 5. Finally, in step 6, a third data subset was used to predict the dengue incidence and determine the accuracy of the method.

Rule extraction from the training data (step 3) is the most important and novel step of the whole methodology. It is performed using FARM, a set of data mining methods that automatically extract from data so-called fuzzy association rules [41]. Fuzzy association rules are of the form:

IF (X is ***A***) → (Y is ***B***).

where X and Y are variables, and ***A*** and ***B*** are fuzzy sets that characterize X and Y respectively. The following is a simple example of a fuzzy association rule (not actually used in the method):

IF (Temperature is ***HOT***) AND (Humidity is ***HIGH***) → (Energy usage is ***HIGH***).

Fuzzy association rules are easily understood by humans because of the linguistic terms that they employ (e.g., ***HOT***, ***HIGH***). Fuzzy set theory [42] assigns a degree of membership between 0 and 1 (e.g., 0.4) to each element of a set, allowing for a smooth transition between full membership (degree=1) and non-membership (degree=0). The degree of membership in a set is generally considered to be the extent to which a corresponding fuzzy set applies. For example, if the variable is temperature and the linguistic term (fuzzy set) is ***HOT*** then you might consider a temperature of 70F to have a degree of membership of 0.1 in the fuzzy set ***HOT***, while a temperature of 80F might have a membership degree of 0.8 and a temperature of 100F might have membership degree equal to 1.

FARM extracts a large number of rules (possibly hundreds or even thousands) from a training data set. When the classifier is automatically built from those rules, the rules have only one consequent, which is the variable to be predicted (i.e., future dengue incidence). When building a classifier, a subset of rules must be chosen; the subset chosen is the one that results in a smallest misclassification error for the validation set. For building the classifier, we have extended the method of Liu et al. [43] as will be described in the section entitled ***Building the classifier***. There are certain class weights that need to be assigned and the final classifier is the one that has the lowest misclassification error on the validation set.

The final step was the computation of predictions by the classifier. The outcome variable (predicted dengue incidence) was converted to a binary variable, either ***HIGH*** or ***LOW*** dengue incidence (where the threshold between high and low values is quantitatively defined in FARM-based methods results section as the mean dengue incidence + 2 standard deviations). Testing was performed on the test data and the following performance metrics were used to assess the accuracy of this prediction: Positive Predictive Value (PPV), Negative Predictive Value (NPV), sensitivity, and specificity. PPV is the proportion of dengue outbreaks that are correctly identified, while NPV is the proportion of periods without outbreaks that are correctly identified.

#### Details of the prediction methodology

##### Association rule mining and fuzzy association rule mining

The goal of data mining is to discover inherent and previously unknown information from data. When the knowledge discovered is in the form of association rules, the methodology is called association rule mining (ARM). An association rule describes a relationship among different attributes. Association rule mining was introduced by Agrawal et al. [44] as a way to discover interesting co-occurrences in supermarket data (the market basket analysis problem). It finds frequent sets of items (i.e., combinations of items that are purchased together in at least *N* transactions in the database), and from the frequent items sets such as {X, Y}, generates association rules of the form: X → Y and/or Y → X. A simple example of an association rule pertaining to the items that people buy together is:

IF (Bread AND Butter) → Milk

The above rule states that if a person buys bread and butter, then they also buy milk. Such rules are very useful for store managers to help decide how to group items on the shelves. Many extracted rules are obvious, as the one mentioned above. However ARM methods extract not only well known rules but, more importantly, novel rules unknown to Subject Matter Experts (SMEs). Those rules are often surprising to SMEs as the now famous rule:

IF Diapers → Beer.

The store managers did not want to believe that there was a relationship between buying diapers and buying beer and thought that the ARM methodology that extracted that rule from data was flawed. However after carefully checking the store transactions, they noticed that in the evenings this rule was very prominent: when somebody was buying diapers they were also buying beer. After further investigation, they concluded that in the afternoons/evenings, moms often ask dads to buy some diapers; dads do that and reward themselves by buying beer.

A limitation of traditional association rule mining is that it only works on binary data (i.e., an item was either purchased in a transaction (1) or not (0)). In many real-world applications, data is either categorical (e.g., district name, type of public health intervention) or quantitative (e.g., rainfall, temperature, age). For numerical and categorical attributes, Boolean rules are unsatisfactory. Extensions have been proposed to operate on these data, such as quantitative association rule mining [45] and fuzzy association rule mining [41].

Fuzzy association rules are of the form:

IF (X is ***A***) → (Y is ***B***).

where X and Y are variables, and ***A*** and ***B*** are fuzzy sets that characterize X and Y respectively. A simple example of fuzzy association rule for a medical application is the following:

IF (Temperature is **S*trong Fever***) AND (Skin is ***Yellowish***) AND (Loss of appetite is ***Profound***) → (Hepatitis is ***Acute***).

**Figure 12:**
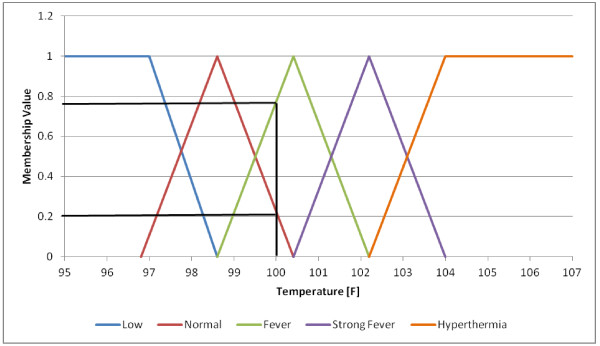
**Membership functions for the fuzzy variable *Temperature*: Low, Normal, Fever, Strong Fever and Hyperthermia.**

The rule states that if a person has a ***Strong Fever***, ***Yellowish*** skin and ***Profound*** Loss of appetite, then the person has ***Acute*** Hepatitis. ***Strong Fever***, ***Yellowish, Profound and Acute*** are membership functions of the variables Temperature, Skin, Loss of appetite and Hepatitis, respectively. As an example of fuzzy membership functions, the membership functions for the variable Temperature are shown in Figure 12. According to the definition in that figure a person with a 100F Temperature has a ***Normal*** temperature with a membership value of 0.2 and has a ***Fever*** with a membership value of 0.78. More information on Fuzzy Logic and fuzzy membership functions can be found in [42].

More formally, let D = {t_1_, t_2_, …, t_n_} be the transaction database and let t_i_ represent the i^th^ transaction in D. Let I={i_1_, i_2_,… i_m_} be the universe of items. A set *X* ⊆ *I* of items is called an itemset. When X has k elements, it is called a k-itemset. An association rule is an implication of the form X → Y, where *X* ⊂ *I*, *Y* ⊂ *I* and *X* ∩ *Y* = *ϕ*.

ARM and FARM rules have certain metrics associated with them. The three metrics most widely used are *Support*, *Confidence* and *Lift* and we will be using these metrics in the method’s development. The *support* of an itemset X is defined as:

$$Support\left(X\right)=\frac{number\;records\;with\;X}{\;number\;records\;in\;D}=\frac{n_{x}}{n}=p_{x}$$

where n is the number of records in D, n_x_ is the number of records with X, and p_x_ is the associated probability. The support of a rule (X → Y) is defined as:

$$Support\left(X\to Y\right)=\frac{number\;records\;with\;X\;and\;Y}{number\;records\;in\;D}=\frac{n_{\mathrm{xy}}}{n}=p_{\mathrm{xy}}$$

where n_xy_ is the number of records with X and Y, and p_xy_ is the associated probability.

The *confidence* of a rule (X → Y) is defined as:

$$Confidence\left(X\to Y\right)=\frac{number\;records\;with\;X\;and\;Y}{number\;records\;with\;X}=\frac{n_{\mathrm{xy}}}{n_{x}}=\frac{n_{\mathrm{xy}}}{n}*\frac{n}{n_{x}}=\frac{p_{\mathrm{xy}}}{p_{x}}$$

Confidence can be treated as the conditional probability (P(Y|X)) of a transaction containing X of also containing Y. A high confidence value suggests a strong association rule. However, this can be deceptive. For example, if the antecedent (X) or consequent (Y) have a high support, they could have a high confidence even if they were independent. This is why the measure of lift was suggested as a useful metric.

The *lift* of a rule (X → Y) measures the deviation from independence of X and Y:

$$Lift\left(X\to Y\right)=\frac{Confidence\left(X\to Y\right)}{Support\left(Y\right)}=\frac{p_{\mathrm{xy}}}{p_{x}}*\frac{1}{p_{y}}=\frac{p_{\mathrm{xy}}}{p_{x}*p_{y}}$$

A lift greater than 1.0 indicates that transactions containing the antecedent (X) tend to contain the consequent (Y) more often than transactions that do not contain the antecedent (X). The higher the lift, the more likely that the existence of X and Y together is not just a random occurrence, but rather due to the relationship between them.

#### Building the classifier

FARM extracts a large set of rules from the training data. For the disease prediction application, the rules of interest are called class association rules (CARs), meaning that they have only one consequent - the class. An example of a CAR extracted by FARM is:

IF (Past_Incidence_Rate_T-1 is ***HIGH***) AND (Past_Incidence_Rate_T-5 is ***HIGH***) AND (Rainfall_T-3 is ***LARGE***) → (Predicted_Incidence_Rate_T+4 is ***HIGH****)*, confidence = 0.95, support = 0.01, lift = 5.3.

The question is which rules from the hundreds extracted by FARM to use in the final classifier and in which sequence to use them. When building the classifier, we first employed the method of Liu et al. [43]. Let *R* be the set of generated rules and *D* be the training data. The basic idea of the algorithm is to choose a subset of rules from *R* to cover all the training examples (*D*). The classifier will have the following format: <r_1_, r_2_, …, r_m_, default class>. Default class is the one into which a case will be classified, if none of the rules satisfies it. The order of the rules in the classifier is important and in classifying a case, the first rule that satisfies it will classify it.

The algorithm has the following steps:

Step 1: Order the rules in *R* by:

1 Confidence (from highest to lowest);

2 Support (from highest to lowest);

3 Number of antecedents (from lowest to highest).

Step 2: Select the rules for the classifier from *R*. For a given rule *r*, find cases in *D* that are covered by *r* (i.e. they satisfy the conditions of *r*). Remove from *D* the cases covered. Compute the number of errors that the rule makes and add the rule to the classifier (*C*). A default class is also selected – this is the majority class in the remaining data in *D*. When there is no rule or no training case left, then the rule selection process is completed.

Step 3: Discard the rules from *C* that do not improve the accuracy of the classifier.

The classifier generated by the algorithm above did not have a satisfactory accuracy for dengue prediction and had the tendency to classify almost all of the cases into the ***LOW*** category. The ***LOW*** category is a majority category in the data and 94.5% of training data are ***LOW***. This makes it much more challenging for the classifier to learn to classify cases as ***HIGH***. Therefore we introduced the following changes to the classifier building algorithm:

1 The rules are being ordered first by confidence, then by lift, and finally by the number of antecedents.

2 The misclassification error is weighted. The user has the opportunity to give a much higher weight for misclassifying the cases that should be ***HIGH*** than those that should be ***LOW***. We used 10 and 1, respectively.

## Results

### FARM-based method results

An example rule extracted by FARM from the data is:

IF (Past_Incidence_Rate_T-1 is ***HIGH***) AND (Past_Incidence_Rate_T-5 is ***HIGH***) AND (Rainfall_T-3 is ***LARGE***) → (Predicted_Incidence_Rate_T+4 is ***HIGH****)*, confidence = 0.95

The above rule states that if the dengue incidence rate a week ago (T-1) was ***HIGH***, the dengue incidence rate five weeks ago (T-5) was ***HIGH***, and the rainfall three weeks ago was ***LARGE***, then the predicted dengue incidence rate in four weeks (T+4) will be ***HIGH***. Each extracted rule has an associated confidence that measures the conditional probability that if the left hand side of the rule was true, the right hand side is also true.

Three different prediction models (classifiers) were automatically built using the methodology developed. The first two are weekly, i.e. we predicted either ***HIGH*** or ***LOW*** dengue incidence for a given future week (T+3 and T+4). The third prediction encompassed a four-week period, specifically four to seven weeks from time of prediction (T+4 to T+7). This is a single prediction for whether dengue incidence rate will be ***LOW*** or ***HIGH*** over the entire four-week period.

In order for a dengue incidence prediction to fall exclusively into one class (***LOW*** or ***HIGH***), we needed to set the threshold between ***LOW*** and ***HIGH***. For weekly data, this was achieved by computing the mean (0.103) and standard deviation (0.175) of past weekly incidences. The threshold between ***LOW*** and ***HIGH*** was set at mean + 2 standard deviations (rounded to 0.45). For 4–week data, the mean was 0.343 and standard deviation was 0.583. The threshold between ***LOW*** and ***HIGH*** was set at mean + 2 standard deviations (rounded to 1.5).

For the weekly incidence data, the predictor variables used were past incidence rate, rainfall, day temperature, night temperature, NDVI, EVI, SSTA, and SOI, where each had 13 weekly values (i.e., weeks T-12, T-11, …, T-1, T). Additional variables were week number, running water, sanitation, and electric lighting. Together this set, including lags, contained 108 variables. Predictor variables were chosen from these 108 variables based on the team’s meteorological and epidemiological experience as well as the quality of a given variable’s data. For example, the weekly predictions did not use the temperature variables because those had a large number of missing values. The variables used for various predictions are shown in Table 3.

**Table 3:** Variables used

**Weekly prediction: Prediction 3 weeks ahead**	**Weekly prediction 4 weeks ahead**	**4 Week prediction 4–7 weeks ahead**
Past Incidence Rate (T-12, T-11, …, T-1, T)	Past Incidence Rate (T-12, T-11, …, T-1)	Past Incidence Rate (T-12_T-9, T-8_T-5, T-4_T-1)
Rainfall (T-12, T-11, …, T-1, T)	Rainfall (T-12, T-11, …, T-1)	Rainfall (T-12_T-9, T-8_T-5, T-4_T-1)
NDVI (T-12, T-11, …, T-1, T)	NDVI (T-12, T-11, …, T-1)	NDVI (T-12_T-9, T-8_T-5, T-7_T-4)
SOI (T)	SSTA (T-4, T-3, T-2, T-1)	EVI (T-12_T-9, T-8_T-5, T-7_T-4)
Week number	Week number	SSTA (T-12, T-11, …, T-1)
		SOI (T-12_T-9, T-9_T-6)
		Temperature Day (T-12_T-9, T-8_T-5, T-5_T-2)
		Temperature Night (T-12_T-9, T-8_T-5, T-5_T-2)
		Week number
		Running water
		Sanitation
		Electric lighting

The weekly, T+3 (i.e., three-weeks in advance) incidence prediction achieved a PPV of 0.667, a NPV of 0.983, a sensitivity of 0.593, and a specificity of 0.987 on the test data set. This means that if the method predicted there would be a ***HIGH*** dengue incidence three weeks in the future, a ***HIGH*** dengue incidence occurred 66.7% of the time and 59.3% of the total ***HIGH*** dengue incidence rates three weeks in the future were captured (sensitivity). Similarly, if the method predicted a ***LOW*** dengue incidence three weeks in the future, a ***LOW*** dengue incidence occurred 98.3% of the time and 98.7% of the total ***LOW*** dengue incidence rates three weeks in the future were captured. The weekly, T+4 predictions were slightly less accurate with a PPV of 0.556, a NPV of 0.973, a sensitivity of 0.469, and a specificity of 0.981.

For the 4–7 weeks in the future predictions the final classifier, obtained by our method, has 166 rules. The top 56 rules have a confidence of 1, and the remaining rules have a confidence ranging from 0.998 to 0.553. While Table 3 lists all the variables used in this prediction, Table 4 provides a sampling of the 166 rules chosen from that classifier for predicting dengue incidence 4–7 weeks ahead. Note that not all the rules are shown due to space limitations. However, the purpose of Table 4 is to help illustrate the method by showing these example rules. As an example let’s consider rule 58:

**Table 4:** Examples of the 166 chosen rules from the classifier for predicting 4–7 weeks ahead

**Rule #**	**Antecedent 1**	**Antecedent 2**	**Antecedent 3**	**Consequent**	**Confidence**	**Support**	**Lift**
1	Week_26-29	PastIncidenceRateT-4_T-1_Low	PastIncidenceRateT-8_T-5_Low	PredictedIncidenceRateT+4_T+7_Low	1.0	0.0454	1.29
2	Week_26-29	PastIncidenceRateT-8_T-5_Low	ElectricLighting_High	PredictedIncidenceRateT+4_T+7_Low	1.0	0.0402	1.29
…	…	…	…	…	…	…	…
46	Week_42-45	PastIncidenceRateT-4_T-1_High	SSTAT-10_High	PredictedIncidenceRateT+4_T+7_High	1.0	0.0047	18.83
…	…	…	…	…	…	…	…
58	NDVIT-8_T-5_Med	Sanitation_High	SSTAT-2_High	PredictedIncidenceRateT+4_T+7_Low	0.998	0.0122	1.29
…	…	…	…	…	…	…	…
166	Week_50-53	RainfallT-12_T-9_Med	SOIT-12_T-9_High	PredictedIncidenceRateT+4_T+7_High	0.553	0.0038	10.41

IF (NDVI_T-8_T-5 is ***MED***) AND (Sanitation is ***HIGH***) AND (SSTA_T-2 is ***HIGH***) → (Predicted_Incidence_Rate_T+4_T+7 is ***LOW****)*, confidence = 0.998, support = 0.0038, lift = 1.29

**Figure 13:**
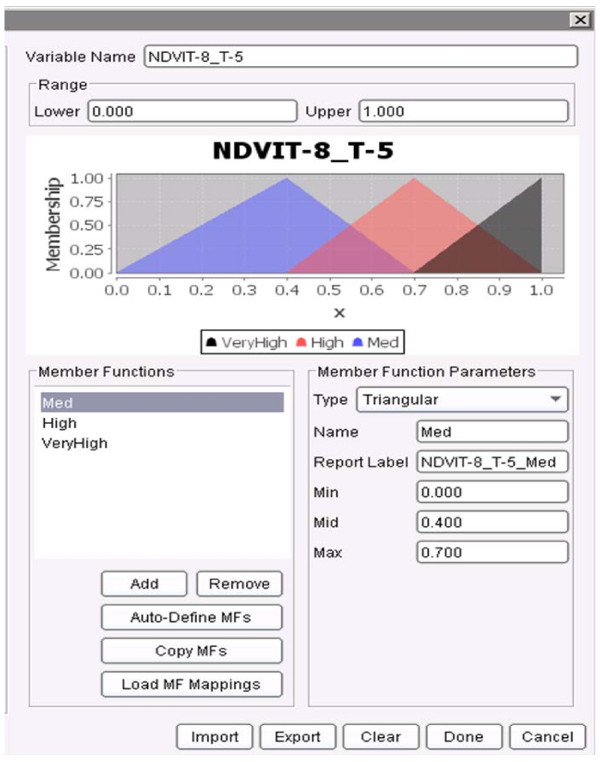
**Membership functions for the variable *NDVI _T-8_T-4 *as defined in the JHU/APL FARM software.** The membership functions are: Med, High and Very High.

**Figure 14:**
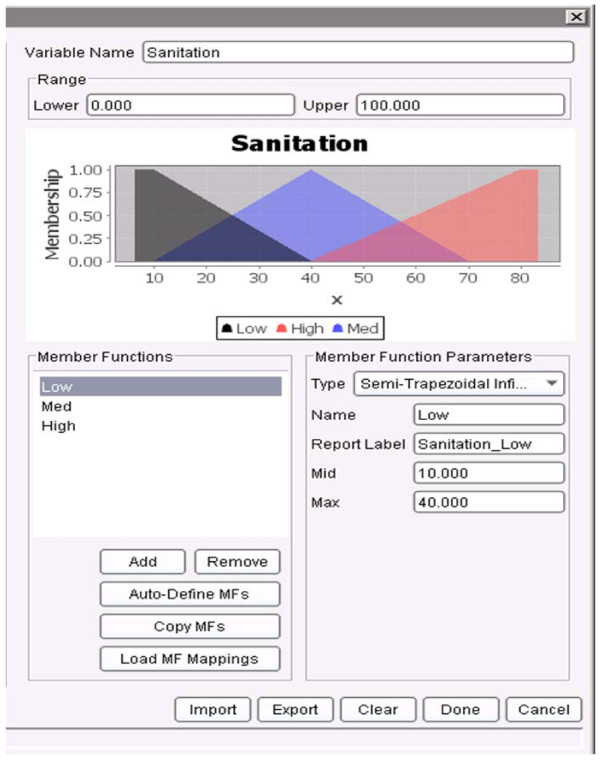
**Membership functions for the variable *Sanitation *as defined in the JHU/APL FARM software.** The membership functions are: Low, Med and High.

**Figure 15:**
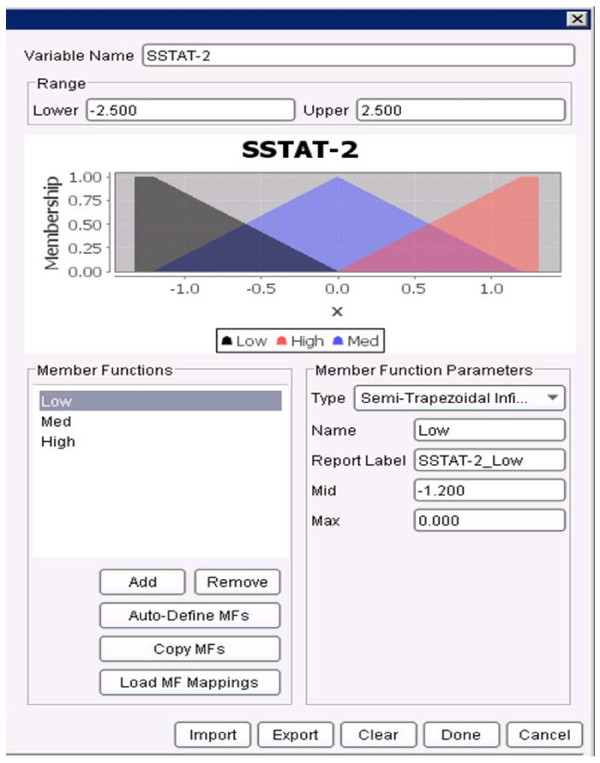
**Membership functions for the variable *SSTA_T-2 *as defined in the JHU/APL FARM software.** The membership functions are: Low, Med and High.

First, the membership functions for all the variables had to be defined. Figure 13, Figure 14 and Figure 15 show the membership functions for antecedents of the rule above: NDVI_T-8_T-5, Sanitation, and SSTA_T-2. The rule states that if the NDVI eight weeks ago to five weeks ago (T-8_T-5) was ***MED*** (i.e., Medium as defined in Figure 13), the Sanitation was ***HIGH*** (as defined in Figure 14), and the SSTA two weeks ago was ***HIGH*** (as defined in Figure 15), then the predicted dengue incidence rate in four weeks to seven weeks (T+4_T+7) will be ***LOW*** (defined earlier as less than the sum of the mean incidence rate plus two standard deviations). The rule has confidence of 0.998 (that measures the conditional probability that if the left hand side of the rule was true, the right hand side is also true), the support of 0.0038 (so it describes 0.38% of the training data) and a lift of 1.29.

**Figure 16:**
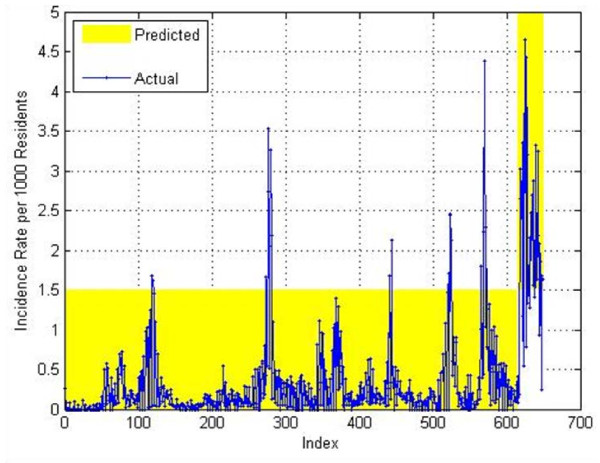
4-Week prediction results for the test set.

**Figure 17:**
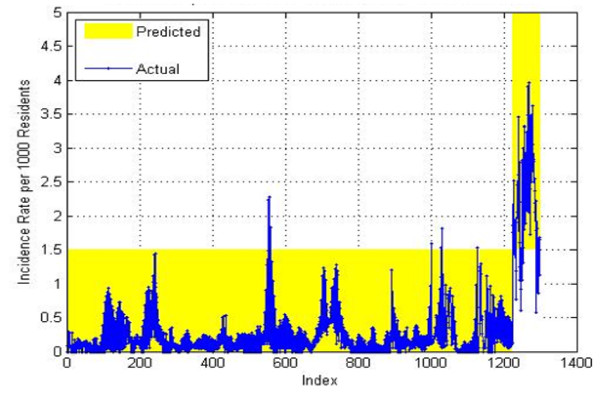
**4-Week prediction results for the training set.** When the blue curve (actual) falls within the yellow bar (prediction) there is no error. When the blue curve falls outside of the yellow bar, there is a prediction error.

**Figure 18:**
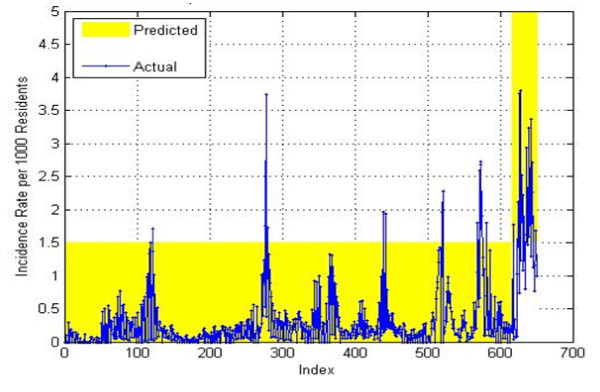
4-Week prediction results for the validation set.

Our overall results on the test data for the 4–7 weeks in the future prediction achieved a PPV of 0.686, a NPV of 0.976, a sensitivity of 0.615, and a specificity of 0.982 and can be seen on Figure 16. The predictions on the training data are shown on Figure 17. For training data a PPV of 0.842, a NPV of 0.996, a sensitivity of 0.928, and a specificity of 0.99 were obtained. As mentioned previously, good accuracy on the training data is relatively easy to achieve since the system has used that data for building the model. The predictions on the validation data are shown on Figure 18. For training data, a PPV of 0.606, a NPV of 0.976, a sensitivity of 0.571, and a specificity of 0.979 were obtained.

**Figure 19:**
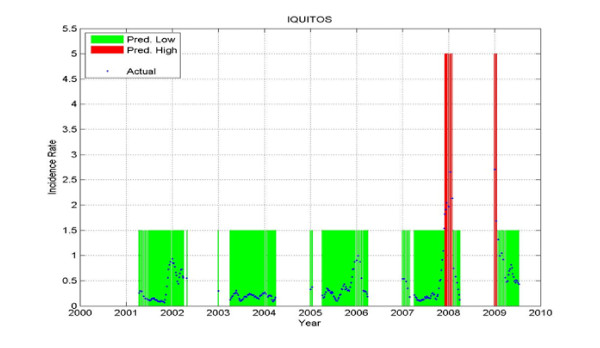
**Prediction on the training data for Iquitos.** The predicted Low is shown in green, the predicted High in red, and the actual values are shown in blue. The gaps with no values correspond to data that was not used in training. When the blue curve (actual) falls within the green bar (predicted Low) or red bar (predicted High) there is no error. When the blue curve falls outside of those bars, there is a prediction error.

**Figure 20:**
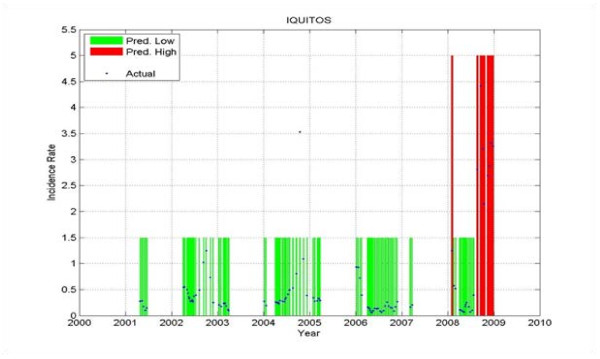
**Prediction on the validation data for Iquitos.** The predicted Low is shown in green, the predicted High in red, and the actual values are shown in blue. The gaps with no values correspond to data that was not used in validation.

**Figure 21:**
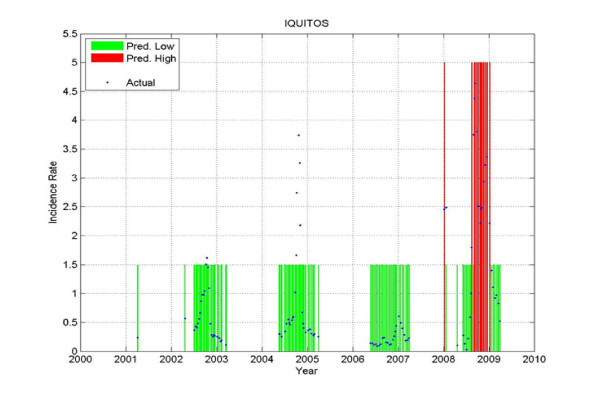
**Prediction on Test data for Iquitos.** The predicted Low is shown in green, the predicted High in red, and the actual values are shown in blue. The gaps with no values correspond to data that was not used in testing.

Figure 19, Figure 20 and Figure 21 show the prediction results for one district only: Iquitos - the district that had the most weeks with ***HIGH*** incidence rate from all the six districts under consideration. The results on the test set are: a PPV of 1, which means that every time a ***HIGH*** value was predicted, an outbreak happened; a NPV of 0.902; a sensitivity of 0.591; and a specificity of 1.

### Logistic regression results

In order to compare the results of the novel technique proposed with those of an established method, we compared FARM results with those of a method often used by epidemiologists: logistic regression (LR). We used the same input data for the LR models as for the FARM methods (see Table 3), for both weekly and four-week interval predictions.

LR is a model used for prediction of the probability of occurrence of an event and it is the method of choice among statisticians when the outcome (Y – the dependent variable) is binary. The goal of LR is to predict the likelihood that Y is equal to 1 given certain values of the independent variables X_1_ through X_k_. The form of the logistic model formula is:

$$p=1/\left(1+\exp\left(-\left(B_{0}+{B_{1}}^{*}X_{1}+{B_{2}}^{*}X_{2}+\ldots +{B_{k}}^{*}X_{k}\right)\right)\right)$$

where p is the probability that Y is 1, B_0_ is a constant (called the intercept), and B_1_ though B_k_ are the coefficients for the predictor variables X_1_ through X_k_.

In our application, the LR result gives the probability that a ***HIGH*** incidence rate will occur. Specifically, if the estimate exceeds a predefined threshold (0.5 in our work), the model predicts a ***HIGH*** incidence rate; otherwise, a ***HIGH*** is not predicted.

For three-weeks in advance (T+3), LR yielded a PPV of 0.5, a NPV of 0.962, a sensitivity of 0.25, and a specificity of 0.987. When predicting four-weeks in advance (T+4), LR obtained PPV of 0.583, a NPV of 0.961, a sensitivity of 0.219, and a specificity of 0.992. The T+4 through T+7 four week prediction using LR achieved a PPV of 0.178, a NPV of 0.949, a sensitivity of 0.205, and a specificity of 0.939. The coefficients obtained for the best of LR models, four-weeks in advance (T+4) prediction, are shown in Table 5.

**Table 5:** LR coefficients obtained for weekly predictions 4 weeks ahead (T+4)

**Variable**	**LR Coefficient**
Intercept	17.37
Week	−0.01
PastIncidenceRate T-1	−2.30
PastIncidenceRate T-2	−3.33
PastIncidenceRate T-3	−1.51
PastIncidenceRate T-4	−0.22
PastIncidenceRate T-5	0.30
PastIncidenceRate T-6	0.46
PastIncidenceRate T-7	2.49
PastIncidenceRate T-8	−0.09
PastIncidenceRate T-9	1.19
PastIncidenceRate T-10	−5.89
PastIncidenceRate T-11	1.19
PastIncidenceRate T-12	0.64
Rainfall T-1	0.01
Rainfall T-2	0.01
Rainfall T-3	0.00
Rainfall T-4	0.00
Rainfall T-5	0.00
Rainfall T-6	−0.01
Rainfall T-7	−0.01
Rainfall T-8	0.00
Rainfall T-9	0.00
Rainfall T-10	0.00
Rainfall T-11	0.00
Rainfall T-12	−0.01
NDVI T-4	0.20
NDVI T-5	3.83
NDVI T-6	−5.59
NDVI T-7	1.86
NDVI T-8	−3.45
NDVI T-9	1.84
NDVI T-10	−5.75
NDVI T-11	−1.99
NDVI T-12	−5.21
SSTA T-1	−2.11
SSTA T-2	1.21
SSTA T-3	−0.79
SSTA T-4	1.16

**Figure 22:**
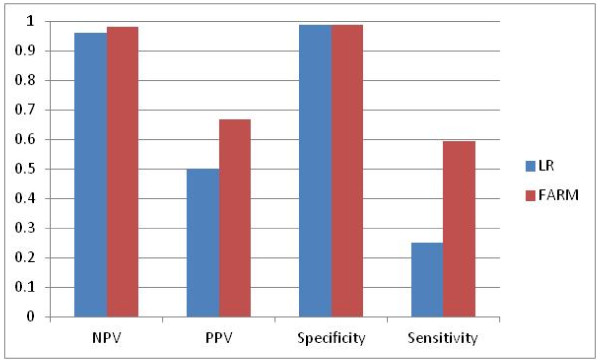
Comparison of LR and FARM-based results for weekly predictions three-weeks in advance (T+3).

**Figure 23:**
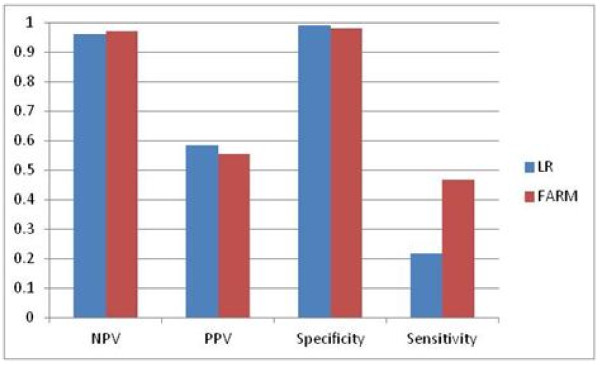
Comparison of LR and FARM-based results for weekly predictions four-weeks in advance (T+4).

**Figure 24:**
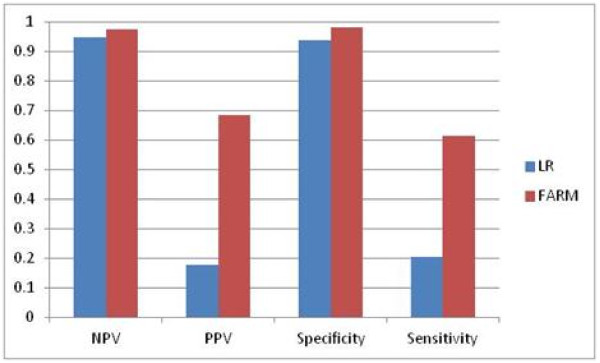
Comparison of LR and FARM-based results for four week interval prediction: weeks T+4 through T+7.

The comparison of LR and FARM-based results is shown in Figure 22, Figure 23 and Figure 24. FARM-based predictions provide much higher sensitivity than LR’s. In 66% of cases FARM provides also much higher PPV than LR.

## Discussion

A truly rigorous predictive method should have two characteristics that cannot be violated. The first one is that the method cannot be both developed and tested on exactly the same data. Rigorous validation requires that the data used for testing not be the same as the data used in its development. If the prediction method was developed and tested on the same data, then a high value of a performance metric, such as R^2^, does not reveal anything about the accuracy that would occur on previously unseen data. Even obtaining R^2^=1 when using the same data for both development and validation does not guarantee a good prediction performance on data not used for the model development.

The second characteristic for rigorous prediction is that all predictor variables need to be collected for the previous time period (e.g. week) and be used for prediction of outbreaks during a later time period. This ensures a realistic prediction because the values of all the predictor variables can be obtained prior to performing prediction for the next time period. Methods that use some variables at time T to predict another variable at time T (i.e., zero time lag) are not performing a useful prediction because prediction means using past or currently available data to describe a future event.

When designing a prediction method that learns from data, machine learning scientists very carefully divide the data set they are using. Simply dividing the data set into training (to develop the model) and testing (to test the model and report performance) is considered insufficient. These data should be divided into three subsets: training, validation and testing [46]. The training subset is used to develop the model. The models are usually not parameter-free, but have certain parameters that can be adjusted by the model developers: the best model is the one that has the lowest error on the validation data subset. Once the best model is chosen, then it becomes the final version and it can be tested to assess its performance on a previously unseen data set called the testing subset. For example, in a feed-forward neural network, the number of hidden layers and the number of neurons in each hidden layer are parameters to be chosen; when several of those networks are compared, the best network has the smallest error on the validation data subset. Once the network with the smallest validation error is chosen, the error is computed for the test subset in order to assess its performance on previously unseen data.

It is important to note that our method only uses input data that is actually available prior to running the model at a given time. For example, for the Temperature variable, we ignored data within two weeks of the current week in order to avoid using values that actually would not be available at the point in time at which the prediction is made. Temperature is provided in 8-day intervals so, if such an interval ends on a Sunday, none of the Temperature values for the preceding week would be available because the interval from which some of their data were coming would not be available until the following Monday.

Disease outbreak detection differs from prediction in that the evidence of the incipient outbreak is already present though not yet obvious when it is first detected, and a response should begin immediately. Although it may complement disease detection, the work presented here differs in that it can be used when no outbreak is currently present, and it predicts whether or not an outbreak may occur at some specific time in the future. Response to such a prediction may include planning as well as mitigation activities. Our method is designed to produce a dengue outbreak prediction four to seven weeks in advance, thereby providing public health officials with more time to intervene and perhaps mitigate the impacts of an outbreak. Discussions with our Peruvian collaborators revealed that this response timeline is reasonable for their public health departments. They did caution however, that it is important to have a method with few false alarms because of limited funding for public health interventions. The parameter of greatest importance to these public health practitioners is therefore PPV, with specificity being second in priority.

Our method has several weaknesses. First, it has no input variables that directly measure vector behavior, e.g., mosquito biting behavior or prevalence of dengue virus in the vector. This information is quite important, yet is expensive and labor intensive to obtain, and possible only for small areas over short time periods. The reason we are not using this variable is that we do not have access to such data for Peru. Also, because the socio-political and sanitation data are only available as annual updates, our method cannot predict the effects of concentrated sanitation programs such as house-to-house efforts to remove vector breeding containers. Another variable that could be useful for prediction are people’s travel patterns. If people were traveling from a district with an ongoing outbreak, these data have the potential to be important predictors. Additionally, we do not have historical data describing the serotypes present and existing dogma supports a role for pre-existing immunity to a serotype with which a person was infected before. Given the fact that the methodology developed herein automatically extracts rules from existing data, the weaknesses described above can be overcome should the data become available.

## Conclusions

The method described above was developed to use local and remote sensing data to predict dengue outbreaks, with an outbreak defined as being above the long-term mean previous dengue incidence plus two standard deviations, with high values of PPV and NPV. Effective methodologies to predict disease outbreaks may allow preventive interventions to avert large epidemics. For best results, the researchers must have access to data streams with timely, detailed, and accurate values of predictor variables. Model validation is of paramount importance as health officials would be unlikely to spend resources on mitigation efforts based on model predictions without evidence of accuracy on past outbreaks. The input variables used in our model are widely available for most, if not all, countries. Although additional local data, such as mosquito biting activity or percentage of mosquitoes with dengue virus, might improve the accuracy of our method, such data are generally difficult and expensive to obtain. The use of widely available data enhances the generalizability of our method.

## Competing interests

The authors’ declare that they have no competing interests.

## Authors’ contributions

ALB directed the project, conceived the prediction methodology used, run the fuzzy association rule mining and classification software to obtain prediction results. She wrote portions of the manuscript and contributed to reviewing of the manuscript. PTK conceived the methods for preprocessing the data to fit the spatiotemporal scale, and implemented all routines in MATLAB to do the preprocessing. He also developed the Logistic Regression Model. He wrote a part of the rough draft of the manuscript. SMB contributed satellite remote sensing (e.g., selection of satellite products) and atmospheric science expertise, as well as medical expertise to the analysis and interpretation of the data. He contributed to the writing of the manuscript. BHF contributed his medical and public health expertise to the interpretation of the data. He wrote portions of the manuscript. SHL was instrumental in acquiring the epidemiological data. She contributed to the writing and reviewing of the manuscript. All authors read and approved the final manuscript.

## Disclaimer

The views expressed here are the opinions of the authors and are not to be construed as official or as representing the views of the US Department of the Navy or the US Department of Defense.

## Pre-publication history

The pre-publication history for this paper can be accessed here:

http://www.biomedcentral.com/1472-6947/12/124/prepub
